# Post-trigger luteinizing hormone concentration to positively predict oocyte yield in the antagonist protocol and its association with genetic variants of LHCGR

**DOI:** 10.1186/s13048-023-01271-6

**Published:** 2023-09-11

**Authors:** Hao Jin, Haiyan Yang, Jiujia Zheng, Jiechun Zhou, Rong Yu

**Affiliations:** 1 The Urological Surgical Department, the First Affiliated Hospital of Wenzhou Medical University, Wenzhou, China; 2 The Reproductive Center, the First Affiliated Hospital of Wenzhou Medical University, Wenzhou, China No. 96, Fuxue Road, Lucheng District,

**Keywords:** Oocyte yield, LHCGR, Trigger, Luteinizing hormone, Human chorionic gonadotropin

## Abstract

**Background:**

The concentration of human chorionic gonadotropin (hCG)/ luteinizing hormone (LH) after triggering is generally accepted as a predictor of the normal ovarian response to the trigger, but few studies have explored the distribution model of concentration and its impact on oocyte yield. Genetic variations in LHCGR, known as a receptor for hCG and LH, also play a role in oocyte maturation and retrieval. The objective of the study was to investigate the impact of concentrations of hCG/LH after triggering on oocyte yield and its association with genetic variants of LHCGR.

**Methods:**

A retrospective cohort study including 372 antagonist IVF cycles, in which 205 received the recombinant hCG trigger and 167 received the gonadotropin-releasing hormone agonist (GnRH-a) trigger, was conducted. The post-trigger concentrations of hCG/LH and the LHCGR N312S (rs2293275) genotype were evaluated in patients to analyse the impact of these factors on oocyte yield.

**Results:**

The oocyte retrieval rate (ORR) increased significantly among the low-, medium- and high-hCG-concentration groups (0.91 ± 0.25, 0.99 ± 0.23 and 1.08 ± 0.19, *P* < 0.001) and among the low-, medium- and high-LH-concentration groups (0.80 ± 0.29, 0.95 ± 0.21 and 1.07 ± 0.19, *P* < 0.001). The Pearson correlation coefficient between the post-trigger hCG concentration and ORR was 0.242 (*P* < 0.001), and that between the LH concentration and ORR was 0.454 (*P* < 0.001). After adjustment for confounding factors, high post-trigger LH concentrations remained associated with the significantly higher ORRs (adjusted R^2^ = 0.541, *P* < 0.001). Patients with the AG genotype of LHCGR N312S were more likely to have low post-trigger LH concentrations (46.10 IU/L versus 60.91 IU/L, *P* < 0.001) and a significantly lower ORR (0.85 versus 0.96, *P* = 0.042) than patients with the GG genotype after the GnRH-a trigger.

**Conclusions:**

The post-trigger LH concentration can positively predict oocyte yield in antagonist IVF cycles, and patients with the AG genotype of LHCGR rs2293275 could have a suboptimal oocyte yield using the GnRH-a trigger.

**Supplementary Information:**

The online version contains supplementary material available at 10.1186/s13048-023-01271-6.

## Background

When oocytes are harvested during IVF treatment, follicles with diameters of more than 10–12 mm are aspirated [1]. The ratio of the expected number of oocytes to the number of follicles aspirated should be close to 1, but precise criteria are lacking. However, sometimes, low oocyte yield occurs even with an experienced physician [2]. This is rare and difficult to interpret because it is not known whether it is due to operational accidents or pathological factors. One extreme condition associated with low oocyte yield is empty follicle syndrome (EFS). Since the first report by Coulam et al. in 1986 [3], the authenticity and prevalence of the disease have been questioned. In many but not all cases, trigger medication is injected incorrectly, known as “false EFS” [4]. In other cases of "genuine EFS" with elevated serum human chorionic gonadotropin (hCG) or luteinizing hormone (LH) levels after triggering, the LHCGR and ZP genes were found to have mutations, most of which were located in exons. It has been reported that variations in the LHCGR gene may lead to receptor defects associated with protein changes in transmembrane domains [5], and mutant ZP genes produce a truncated ZP protein, resulting in obstacles to zona pellucida assembly [6]. Patients with genuine EFS are mostly advised to receive donor oocytes, but researchers previously reported that a small number of patients with homozygous mutations of LHCGR had obtained oocytes and pregnancies by delayed follicle aspiration (up to 48 h after trigger) and high doses of trigger medication [7].

The "trigger" step in IVF replicates the preovulation LH surge of the natural cycle [8], which is essential for oocyte maturation, prompting the oocyte to resume meiosis and acquire competence for fertilization. The two triggers commonly used in the last few decades, hCG and gonadotropin-releasing hormone agonist (GnRH-a), act through different mechanisms; specifically, GnRH-a stimulates the pituitary to release LH, whereas hCG directly binds to the LH/hCG receptor. Moreover, hCG has a longer half-life and is therefore associated with more intense stimulation of the receptor than GnRH-a [9]. Although it has been reported that there is no difference in the number of MII oocytes obtained using hCG and agonist triggers in nondonor IVF cycles [10], a few studies have presented a higher incidence of low oocyte yield in GnRH-a trigger cycles [11]. In particular, low serum LH levels after the GnRH-a trigger strongly indicate oocyte recovery failure [12]. Castillo et al. observed no difference in the incidence of EFS between the GnRH-a (3.5%) and hCG (3.1%) triggers [13], suggesting that low oocyte yield related to suboptimal response to trigger may have more to do with personal characteristics than trigger types.

Endogenous LH and hCG stimulate the same receptor, LHCGR [14], which belongs to the G protein-coupled receptor family and is distributed in the theca cells of the antral follicle and the granulosa cells of the peri-ovulatory follicle [15]. With the development of pharmacogenetics, several variants of gonadotropin receptors have been proposed to be associated with the ovarian response and the sensitivity of receptors to ligands [16]. In addition to the EFS induced by homozygous mutations of LHCGR mentioned above, researchers found that the presence of serine at LHCGR N312S might lead to reduced sensitivity to LH and heralded more r-hLH supplements during ovarian stimulation for carriers of this polymorphism than noncarriers [17]. Serine carriers were also thought to require a higher follicle-stimulating hormone (FSH) dosage than asparagine carriers in IVF because polymorphisms of LHCGRs could also reduce ovarian sensitivity to FSH [18]. The effect of N312S variants on pregnancy outcomes remains controversial in different populations. Guo [19] demonstrated that carriers of S312 in *LHCGR* more often became pregnant in IVF than those with N312, but data from patients from multiple ethnic groups suggested that LHCGR N312S was not associated with IVF outcomes and should not be considered a reproductive predictor in 1183 PGT-A cycles [20].

To the best of our knowledge, reports on the effect of genetic variation of LHCGR N312S (rs2293275) on oocyte retrieval efficiency in follicle aspiration are rare. Although it is recognized that post-trigger hCG or LH levels are important for successful oocyte recovery, it is not clear whether the expected oocyte yield can be achieved if the hCG or LH levels exceed the commonly used threshold. Since there is no routine test, the prevalence of polymorphisms in LHCGR N312S and whether they impact post-trigger hormone concentrations are unknown. The objective of the current study was to investigate the relationship between the genotypes of LHCGR N312S and the concentrations of hCG/LH after triggering and whether the oocyte yield was affected.

## Methods

### Study population

In total, 372 women who underwent their first IVF cycles during which they completed oocyte recovery from February 2020 to July 2021 were consecutively enrolled. The inclusion criteria were as follows: Chinese Han ethnicity, non-smoker, aged less than 35 years, body mass index (BMI) = 16–28 kg/m^2^, menstrual cycle length = 25–35 days, AMH ≥ 1.5 ng/ml, ovarian stimulation performed with the GnRH antagonist protocol, and more than five follicles with a diameter above 12 mm before aspiration. The exclusion criteria were hypothalamus or pituitary dysfunction, thyroid dysfunction, endometriosis, chromosomal abnormalities or hereditary disease; LH concentration ≥ 10 IU/L before trigger; and use of coasting. The study was approved by the ethical committee board of the affiliate hospital. Written informed consent was obtained from all participants. This was a retrospective study without any intervention in terms of clinical therapy from the researchers.

### IVF treatments

The antral follicle count (AFC) was obtained by counting the number of follicles with diameters of 2–9 mm on days 2–4 of natural menstruation or withdrawal bleeding by transvaginal ultrasound, and basal FSH, LH and oestradiol (E2) concentrations were evaluated in peripheral blood. Ovarian stimulation was initiated with either recombinant FSH (r-FSH, Serono, Germany) or human urine-derived FSH (urofollitropin, Livzon, China) at a daily dose of 150–250 IU, per the patient's age, AFC, and BMI. On days 5–6 of stimulation, the gonadotropin dose was adjusted as needed after ultrasound monitoring, and daily administration of GnRH antagonist (Cetrorelix Acetate, Serono, Switzerland) 0.25 mg was added. Afterwards, follicle size was measured every 2–3 days until at least two dominant follicles reached a diameter of 18 mm, the concentrations of LH, E2 and progesterone (P) in peripheral blood were assessed, and either 250 μg of recombinant hCG (r-hCG, Serono, Switzerland) or 0.2 mg of GnRH-a (Triptorelin, Ferring Pharmaceuticals, France) was used as the trigger. In cases with a high risk of ovarian hyperstimulation, GnRH-a was the most commonly used trigger in our practice. The levels of serum hCG (for hCG injections) or LH (for GnRH-a injections) were measured in the morning of the second day (10–13 h after trigger was given) as routine in our clinic. Patients signed the consent and were informed that the blood samples would be kept for subsequent detection of LHCGR variants. Patients with concentrations below 10 IU/L were considered to have incorrectly injected the trigger and advised to repeat the trigger step, their oocyte retrieval was delayed, and they were excluded from the study so that no cycles with rescue injections would be included in the analyses. Cycles with a dual trigger were also excluded from the study to avoid effects due to interactions between different drugs.

Oocyte retrieval was conducted 36–38 h after trigger under transvaginal ultrasound guidance. Follicles larger than 12 mm in diameter in the bilateral ovaries were aspirated using a 17G needle (COOK, USA) with a negative pressure of 120 mmHg. During the study period, all punctures were performed by the same highly experienced physician. The follicular fluid was immediately checked by an experienced embryologist under a stereomicroscope (Olympus SZX10, Japan) at a magnification of 6–10 times. The number of oocytes with intact zona pellucida in the cumulus complexes (OCCs) was recorded. OCCs were transferred to Vitrilife G-IVF™ (Vitrilife Sweden, Sweden) medium for further fertilization and culture following our standard clinical practice [21]. The embryos were transferred either 3 or 5 days after oocyte retrieval, while all available embryos for patients who were not suitable for fresh transfer were completely frozen. Embryos were graded at the cleavage stage using the single-step scoring approach described by Machtinger [22]. A high-quality embryo was defined as an embryo having at least seven cells generated from cleavage in 3 days, with a generally uniform size and morphology and less than 20% debris. An available embryo was defined as having more than five cells and a debris of less than 50%. The oocyte retrieval rate (ORR) and mature oocyte retrieval rate (MORR) were defined as the ratio of the number of collected oocytes and mature oocytes to the number of follicles measuring ≥ 12 mm before trigger, respectively. The follicle output rate (FORT) was defined as the ratio of the number of follicles measuring ≥ 12 mm before trigger to the number of antral follicles at baseline.

### Hormone measurement

Serum hormone concentrations were measured using chemiluminescent immunoassay (Beckman, CA, USA) in the central clinical chemistry laboratory at the affiliated hospital. The detection ranges were 0.20–150 IU/L for FSH, 0.20–250 IU/L for LH, 20–10900 pg/ml for E2, 0.10–50 ng/ml for P and 1.2–250,000 IU/L for hCG. The sensitivities of the assay were 0.02 IU/L for FSH, 0.02 IU/L for LH, 5 pg/ml for E2, 0.01 ng/ml for P and 0.05 IU/ml for hCG. The intra-assay and inter-assay coefficients of variation were 3.0 and 6.0% for FSH, 3.0 and 6.0% for LH, 4.0 and 6.0% for E2, 4.0 and 6.0% for P, and 3.0 and 5.0% for hCG, respectively.

### Genotyping of LHCGR N312S

The genetic assessment for LHCGR N312S was performed after the patients had finished IVF and had been enrolled in the study cohort. DNA was extracted from peripheral blood with the Blood Genomic DNA Extraction Kit (Omega, USA) following the manufacturer’s recommendations. After determination of the purity and concentration of DNA with an ultraviolet spectrophotometer (Macy, China), the N312S polymorphism of LHCGR was analysed by polymerase chain reaction (PCR) and Sanger sequencing. Briefly, the PCRs were performed in a reaction system of 30 µl containing 10 μl of double distilled H_2_O, 15 μl of Master Mix (2 × MasterMix, Thermo Scientific, USA), 1 μl of each primer (Tsingke Biotechnology, China), and 120 ng template DNA. The forward primer sequence for LHCGR N312S was 5’-TGTTGACCATGTGACTAGGGA-3’, and the reverse primer sequence was 5’-ACTCTCTCCTCAGGAAGCAT-3’, as designed in the previous literature [18]. The amplification conditions were as follows: denaturation at 96 °C for 2 min; 35 consecutive cycles of denaturation at 96 °C for 30 s, annealing at 61 °C for 30 s, and elongation at 72 °C for 30 s; and a final elongation at 72 °C for 5 min. The PCR product was purified and sequenced on an Applied Biosystems Genetic Analyser (ABI3500, Applied Biosystems, USA).

### Statistical analysis

The distribution of genotype and allele frequencies of LHCGR N312S in Hardy–Weinberg equilibrium was tested by chi-squared analysis. The Shapiro‒Wilk test for normal distribution was performed on quantitative variables, which are shown as the mean (SD), while count data are shown as frequencies and percentages. According to the tertile of serum hCG (or LH) level after trigger, patients were divided into high-, medium- and low-concentration groups. Age, BMI, baseline hormone concentrations, AFC, days of ovarian stimulation, total gonadotropin dose, days of antagonist use, hormone concentrations before and after the trigger, number of oocytes, maturation rate, fertilization rate, FORT, ORR, MORR, number of high-quality embryos and number of available embryos among different groups were calculated and compared by analysis of variance (ANOVA) and the least significant difference (LSD) post hoc test. Genotype frequencies of LHCGR N312S and the proportion of patients with PCOS among the three groups were compared by chi-squared analysis or Fisher's exact test. Pearson correlation coefficients with adjustment for BMI [23] were used to analyse the association of post-trigger hCG (or LH) concentration with ORR. A linear regression analysis was conducted to clarify the impact of hCG (or LH) concentration on ORR with adjustments for possible confounding factors (age, BMI, basal LH level, days of ovarian stimulation and dose of gonadotrophin used).

To analyse the impact of genotypes of LHCGR N312S on ORR and post-trigger hormone concentrations, patients with the same triggering strategies were further grouped by genotypes of LHCGR N312S. The BMI, days of ovarian stimulation, total gonadotropin dose, post-trigger hCG (or LH) concentrations, oocyte yield, including ORR and MORR, as well as the fertilization rate and the number of high-quality embryos of patients, were compared between groups of different genotypes of LHCGR by independent t tests. Statistical analyses were performed using Statistical Package for Social Sciences version 22.0 (SPSS, IBM, USA) and GraphPad Prism 7.0 (GraphPad Software, Inc., USA). *P* values of < 0.05 were considered statistically significant.

## Results

Oocytes were obtained from all 372 patients with no empty follicle syndrome observed. Allele frequencies for LHCGR N312S were 92.9% for the G allele and 7.1% for the A allele, and they were in Hardy–Weinberg equilibrium with  χ^2^ = 0.48 (*P* > 0.05). The genotype frequencies for the GG, AG and AA genotypes were 86.0% (320/372), 13.7% (51/372), and 0.3% (1/372), respectively. In one patient with a post-trigger LH concentration of 33.53 IU/L, oocytes were not obtained after aspiration of 5–6 follicles in one ovary during the planned operation, so aspiration of the remaining follicles was postponed 5–6 h after adequate discussion and notification. In this patient, 6 oocytes were eventually retrieved from 23 preovulatory follicles, and her LHCGR N312S genotype was heterozygous.

Of the 372 patients included in the analysis, 205 received an hCG trigger. Their post-trigger hCG concentrations ranged from 13.32 to 221 IU/L, and their ORRs ranged from 0.25 to 1.5. Among these patients, one had the AA genotype for LHCGR N312S, 180 had the GG genotype, and 24 had the AG genotype. Among the 167 patients who received the GnRH-a trigger, the post-trigger LH concentrations ranged from 10.58 to 182.63 IU/L, and the ORRs ranged from 0.12 to 1.44. In total, 140 patients had the GG genotype, and 27 patients had the AG genotype. Descriptive data of the patients is presented in Supplemental Table 1. The results of the chi-square test suggested no significant difference in the proportion of patients with PCOS or any other infertility causes among the three concentration groups, but there was a significant difference in the proportion of patients with GG genotype and AG genotype.

The ANOVA-LSD test results showed that in both the hCG (Table 1) and GnRH-a trigger cycles (Table 2), the group with a high hormone concentration after trigger had a lower BMI, fewer ovarian stimulation days and a lower gonadotropin dose than the other two groups, with the difference being statistically significant (*p* < 0.001). In addition, the basal LH concentration from patients with the GnRH-a trigger varied significantly among the three post-trigger LH concentration groups, but the difference between patients with the hCG trigger was not significant. Regardless of whether the hCG or GnRH-a trigger was used, there were no significant differences with respect to female age, AMH level, AFC, basal FSH level, days of antagonist use, hormone concentrations on the trigger day, number of aspirated follicles, FORT, maturation rate, fertilization rate, and number of high-quality embryos among groups of different post-trigger hormone concentrations. In terms of treatment outcomes, the high concentration group had significantly higher numbers of oocytes retrieved and available embryos compared to the low concentration group in cycles with GnRH-a trigger. There was no significant difference in clinical pregnancy rates of fresh transfer after hCG-trigger among the groups, but due to a small number of fresh transfers, statistical analysis of pregnancy outcome was precluded on patients with GnRH-a trigger.

**Table 1:** Clinical characteristics and genotype frequencies of LHCGR rs2293275 variations in 205 patients triggered with r-hCG 250 μg

Characteristics	hCG concentration after trigger			*P* value
	hCG < = 80.6 IU/L (*n* = 70)	80.6 IU/L < hCG < = 102.8 IU/L (*n* = 67)	hCG > 102.8 IU/L (n = 68)	
Female age (years)	30.15 (3.38)	30.01 (3.36)	30.65 (3.21)	0.496
Female BMI (kg/m^2^)	22.95 (2.99)	22.54 (3.02)	21.41 (2.63)	0.006
Basal hormone concentrations				
FSH (IU/L)	7.00 (1.87)	7.60 (1.85)	7.32 (2.03)	0.190
LH (IU/L)	4.49 (2.96)	5.32 (3.25)	5.60 (4.14)	0.151
AMH (ng/ml)	4.47 (2.63)	4.08 (1.78)	3.88 (2.01)	0.271
AFC (n)	20.77 (10.75)	18.78 (8.53)	18.78 (8.47)	0.351
Days of gonadotrophin (d)	10.30 (1.82)	10.15 (1.42)	9.56 (1.61)	0.020
Total dose of gonadotrophin (IU)	2081.79 (852.96)	1743.28 (638.97)	1611.58 (441.49)	< 0.001
Days of antagonist (d)	4.97 (1.39)	4.75 (1.16)	4.63 (1.15)	0.264
Hormone concentrations on the trigger day				
LH (IU/L)	3.17 (2.33)	2.67 (1.98)	2.96 (2.04)	0.380
E2 (pg/ml)	2576.07 (1324.02)	2789.03 (1693.88)	2373.03 (1162.01)	0.232
P (ng/ml)	0.95 (0.41)	0.98 (0.50)	0.97 (0.62)	0.934
No. of follicles (> = 12 mm) (n)	13.44 (5.07)	12.57 (4.91)	12.57 (4.35)	0.466
No. of oocytes retrieved (n)	12.16 (5.85)	12.48 (5.87)	13.43 (4.55)	0.369
Mature oocyte rate (× 100%)	0.84 (0.21)	0.84 (0.17)	0.87 (0.12)	0.563
Follicle output rate (× 100%)	0.78 (0.40)	0.77 (0.36)	0.78 (0.37)	0.966
Oocyte retrieval rate (× 100%)	0.91 (0.25)	0.99 (0.23)	1.08 (0.19)	< 0.001
Mature oocyte retrieval rate (× 100%)	0.76 (0.29)	0.84 (0.23)	0.93 (0.18)	< 0.001
Fertilization rate (× 100%)	0.68 (0.21)	0.70 (0.19)	0.75 (0.16)	0.088
No. of high-quality embryos (n)	3.23 (2.50)	3.78 (2.46)	3.88 (2.48)	0.252
No. of available embryos (n)	6.37 (4.88)	6.34 (3.70)	7.62 (3.38)	0.113
Clinical pregnancy of fresh transfer (n)	19/36	23/40	11/27	0.396^a^
LHCGR rs2293275 genotype				
GG	59	59	62	0.602^a^
AG	10	8	6	
AA	1	0	0	

*BMI* Body mass index, *FSH* Follicle stimulating hormone, *LH* Luteinizing hormone, *AMH* Anti-mullerian hormone, *AFC* Antral follicle count, *E2* Oestradiolk, *P* Progesterone, *hCG* human chorionic gonadotropin

^a^*P* values for chi-square or Fisher’s exact tests

**Table 2:** Clinical characteristics and genotype frequencies of LHCGR rs2293275 variations in 167 patients triggered with triptorelin 0.2 mg

Characteristics	LH concentration after trigger			*P* value
	LH < = 44.34 IU/L (*n* = 56)	44.34 IU/L < LH < = 64.74 IU/L (*n* = 56)	LH > 64.74 IU/L (*n* = 55)	
Female age (years)	29.79 (2.97)	28.85 (3.36)	29.45 (3.21)	0.290
Female BMI (kg/m^2^)	23.14 (3.34)	22.75 (2.91)	20.40 (2.22)	< 0.001
Basal hormone concentrations				
FSH (IU/L)	6.76 (1.75)	7.45 (1.76)	7.44 (1.88)	0.072
LH (IU/L)	5.42 (3.12)	5.44 (2.53)	7.01 (4.66)	0.028
AMH (ng/ml)	7.68 (3.84)	8.01 (3.74)	8.17 (4.08)	0.798
AFC (n)	29.11 (10.70)	30.32 (12.34)	29.38 (7.91)	0.814
Days of gonadotrophin (d)	10.41 (1.82)	10.11 (1.41)	9.49 (1.25)	0.006
Total dose of gonadotrophin (IU)	1794.42 (608.95)	1578.35 (474.48)	1222.50 (272.03)	< 0.001
Days of antagonist (d)	5.38 (1.60)	5.16 (1.22)	5.07 (0.94)	0.444
Hormone concentrations on the trigger day				
LH (IU/L)	2.89 (2.29)	3.27 (2.09)	3.22 (2.29)	0.624
E2 (pg/ml)	5604.81 (2687.45)	6049.21 (2546.02)	5941.08 (2362.00)	0.628
P (ng/ml)	1.32 (0.58)	1.51 (0.79)	1.24 (0.47)	0.073
No. of follicles (> = 12 mm) (n)	24.64 (9.05)	25.20 (8.18)	24.75 (8.67)	0.937
No. of oocytes retrieved (n)	19.59 (9.67)	23.88 (9.13)	26.62 (11.90)	0.002
Mature oocyte rate (× 100%)	0.86 (0.15)	0.82 (0.14)	0.82 (0.13)	0.222
Follicle output rate (× 100%)	0.94 (0.47)	0.94 (0.40)	0.87 (0.28)	0.580
Oocyte retrieval rate (× 100%)	0.80 (0.29)	0.95 (0.21)	1.07 (0.19)	< 0.001
Mature oocyte retrieval rate (× 100%)	0.68 (0.27)	0.77 (0.21)	0.87 (0.21)	< 0.001
Fertilization rate (× 100%)	0.69 (0.23)	0.77 (0.15)	0.70 (0.18)	0.071
No. of high-quality embryos (n)	5.39 (3.62)	7.11 (4.56)	7.38 (5.52)	0.051
No. of available embryos (n)	10.32 (5.86)	13.68 (6.84)	14.13 (7.18)	0.005
Clinical pregnancy of fresh transfer (n)	1/1	0/0	1/1	–-
LHCGR rs2293275 genotype				
GG	40	48	52	0.003^a^
AG	16	8	3	
AA	0	0	0	

*BMI* Body mass index, *FSH* Follicle stimulating hormone, *LH* Luteinizing hormone, *AMH* Anti-mullerian hormone, *AFC* Antral follicle count, *E2* Oestradiol, *P* Progesterone

^a^*P* values for chi-square or Fisher’s exact tests

**Fig. 1:**
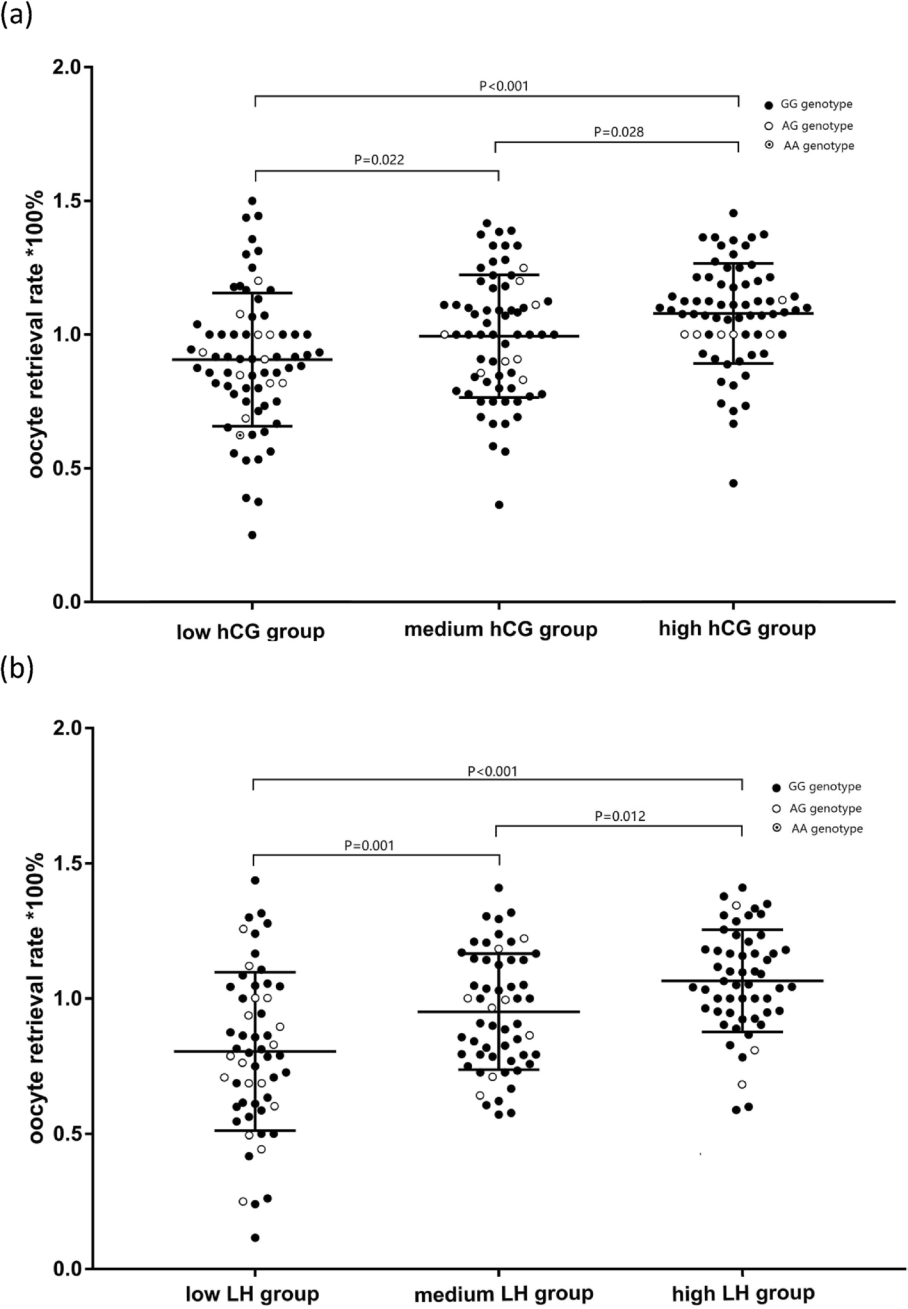
The oocyte retrieval rates in groups of low-, medium- and high-concentrations of hormone after triggering and the genotypes of LHCGR N312S. **a** The oocyte retrieval rate (ORR) increased with increasing serum hCG levels after r-hCG trigger, with significant differences among the low-, medium- and high-concentration groups (*P* < 0.001). The ORRs were 0.91 ± 0.25, 0.99 ± 0.23 and 1.08 ± 0.19, respectively. The points with different shadows present the distribution of LHCGR N312S genotypes. **b** The oocyte retrieval rate (ORR) increased with increasing serum LH levels after GnRH-a trigger, with significant differences among the low-, medium- and high-concentration groups (*P* < 0.001). The ORRs were 0.80 ± 0.29, 0.95 ± 0.21 and 1.07 ± 0.19, respectively. The points with different shadows present the distribution of LHCGR N312S genotypes

Figure 1 presents the distribution of LHCGR N312S genotypes and the significantly increased ORR as the post-trigger hormone concentrations increased among groups (*P* < 0.001). In the low-, medium- and high-hCG groups, the ORRs were 0.91 ± 0.25, 0.99 ± 0.23 and 1.08 ± 0.19, respectively, and in the low-, medium- and high-LH groups, the ORRs were 0.80 ± 0.29, 0.95 ± 0.21 and 1.07 ± 0.19, respectively. After adjustment for BMI, the Pearson correlation coefficient between the post-trigger LH concentration and ORR was 0.454 (*P* < 0.001), and the coefficient between the hCG concentration and ORR was 0.242 (*P* < 0.001). The linear regression analysis showed a significant correlation between post-trigger LH concentrations and ORR (adjusted R^2^ = 0.541, *P* < 0.001), after adjusting for female age, BMI, basal LH level, days of stimulation and dose of gonadotrophin. However, the correlation between post-trigger hCG concentrations and ORR was weak but still significant (adjusted R^2^ = 0.215, *P* < 0.001). To further explore the characteristics of cycles with low oocyte yield, a sub-analysis was conducted on quartile groups of ORR in paitients triggered by GnRH-a. The results suggested that patients with higher ORR had significantly higher post-trigger LH concentrations and number of high-quality embryos than those with lower ORR, while BMI was comparable, as shown in Supplemental Fig. 1.

The frequencies of the GG, AG and AA genotypes of LHCGR N312S (Tables 1 and  2) were not significantly different among the low-, medium- and high-hCG-concentration groups using Fisher's exact test (*P* = 0.602). In contrast, the frequencies of the GG and AG genotypes were significantly different among the low-, medium- and high-LH-concentration groups (*P* = 0.003). When patients were grouped by genotype of LHCGR (Table 3), they had comparable BMI, ovarian stimulation days, total gonadotropin dose and fertilization rates. The average concentrations of hCG after trigger in GG and AG patients were 93.72 ± 30.83 IU/L and 90.00 ± 39.95 IU/L, and the average concentrations of LH after trigger in GG and AG patients were 60.91 ± 28.37 IU/L and 46.10 ± 15.70 IU/L, respectively. The results of the t test showed that there were significantly lower post-trigger LH concentrations, lower ORRs and MORRs, and marginally significantly fewer high-quality embryos in patients with the AG genotype than in patients with the GG genotype during the cycles of the GnRH-a trigger. However, there was no difference in the post-trigger hCG concentration, oocyte yield or number of high-quality embryos between AG and GG genotype patients during the cycles of the hCG trigger. The one patient with the AA genotype was not included in the t test.

**Table 3:** Ovarian stimulation outcomes based on the genotypes of the LHCGR gene in patients triggered with r-hCG and GnRH-a

	r-hCG trigger (*n* = 204)		*P* value	GnRH-a trigger (*n* = 167)		*P* value
Stimulation parameters and outcomes	GG genotype (*n* = 180)	AG Genotype (*n* = 24)		GG genotype (*n* = 140)	AG genotype (*n* = 27)	
BMI	22.28 (2.89)	22.24 (3.25)	0.943	22.06 (3.09)	22.36 (3.21)	0.639
Days of gonadotrophin (d)	10.01 (1.69)	10.00 (1.38)	0.975	9.92 (1.48)	10.44 (1.83)	0.109
Total dose of gonadotrophin (IU)	1825.76 (709.98)	1727.08 (584.92)	0.515	1503.03 (497.47)	1692.13 (644.78)	0.088
Post-trigger hCG (LH) concentration (IU/L)	93.72 (30.83)	90.00 (39.95)	0.593	60.91 (28.37)	46.10 (15.70)	< 0.001
ORR (× 100%)	0.99 (0.24)	0.98 (0.15)	0.674	0.96 (0.26)	0.85 (0.26)	0.042
MORR (× 100%)	0.84 (0.25)	0.82 (0.23)	0.683	0.79 (0.25)	0.69 (0.19)	0.042
Fertilization rate (× 100%)	0.71 (0.19)	0.73 (0.20)	0.521	0.72 (0.19)	0.71 (0.19)	0.775
No.of high-quality embryos (n)	3.55 (2.50)	4.21 (2.41)	0.224	6.89 (4.82)	5.26 (3.69)	0.053

*BMI* Body mass index, *ORR* Oocyte retrieval rate, *MORR* Mature oocyte retrieval rate, *r-hCG* Recombinant human chorionic gonadotropin, *GnRH-a* Gonadotropin-releasing hormone agonists

## Discussion

This study reveals the association between serum hCG (or LH) levels at 10–13 h after trigger and oocyte yield in patients undergoing the antagonist cycle in IVF. High levels of hCG (or LH) predict significantly higher ORR and MORR compared with low—medium levels. The patients involved in the study all had post-trigger hormone levels above 10 IU/L, and this ensured that cases of incorrect trigger administration were excluded. When post-trigger hormone levels below 10–15 IU/L were recognized as a suboptimal reaction to the trigger by most clinicians [24], patients with a hormone concentration above this level were generally considered to be able to obtain satisfactory oocyte yields and did not attract much attention. In a prospective cohort study of 91 patients divided into groups according to each 15-IU increase in LH concentration after trigger, there was a trend towards higher oocyte yield with no linear association in the population with LH >  = 30 IU/L [25]. Another retrospective study of 499 antagonist cycles declared little to no association between the levels of LH-like activity and the number of mature oocytes from different trigger stratagems [26]. The differences in sample size and grouping method should contribute to the difference between our findings and those of others.

In our study, significant differences were present in both the comparisons of patients grouped by post-trigger hormone levels in the tri-sectional quantiles and the analysis of the correlation, suggesting a definite link between hormone levels and oocyte yield; that is, a low level of LH-like activity might be sufficient for effective oocyte recovery in some women, but the recovery would be more reliable with a higher level of LH (or hCG). The correlation coefficient between LH and oocyte yield was observed to be higher than that of hCG, indicating that LH level after GnRH-a trigger could better predict the outcome of oocyte recovery, possibly because of the strong affinity of hCG for the receptor [27], which enables effective receptor activation even with a low level of ligand. Compared with hCG triggering or even the natural cycle, the duration of LH peak after GnRH-a trigger is shorter. This makes the concentration of LH more critical for oocyte recovery, which may explain why our results show a stronger relationship between the LH level and ORR. Notably, the number of high-quality embryos also increased with the hormone level after the trigger, with marginal significance in patients with the GnRH-a trigger (P = 0.051) in this study. This highlights the need for concern for patients with hormone levels slightly above the traditional threshold after the GnRH-a trigger, as they might subsequently experience low numbers of embryos and adverse IVF outcomes. We observed no differences in maturation or fertilization rates among the post-trigger hormone-level groups, which was consistent with the results of other studies [25, 28].

A post-trigger LH concentration of 10–15 IU/L is traditionally used as the standard in many studies to determine whether the ovaries have responded well to the trigger and as an alert that a rescue trigger is needed [24, 29]. However, the lowest ORR observed in our study was 0.12, and the corresponding patient's LH concentration after the GnRH-a trigger was 17.24 IU/L, suggesting that the use of a fixed post-trigger hormone concentration to predict ovarian response may not be appropriate for all patients. A rise in hormone levels could theoretically be considered a measure of success triggering [28], but it is difficult to determine one universal threshold for the magnitude and peak of the rise. For instance, Shapiro et al. demonstrated that the post-trigger LH levels < 52.0 IU/L were suboptimal with the risk of submaximal oocyte yield and maturity, which was obviously different from conventional practice [30]. The reported varying times to peak LH-like activity of various triggers [26] could also contribute to the uncertainty of post-trigger hormone thresholds.

This study enrolled patients with traditionally considered optimal resposes (i.e. LH > 10 IU/L), and we reported the same factors that have been found in other studies to induce a suboptimal response to GnRH-a triggr (i.e. LH < 10-15 IU/L). Our results showed that patients with either hCG or GnRH-a trigger trended to requiring more total gonadotropins and longer stimulation periods when they had lower levels of post-trigger hormones. Additionally, in the cycles of GnRH-a trigger, patients with lower post-trigger hormone levels were found to have significantly lower basal LH levels on the first day of stimulation. These findings suggest that even if the post-trigger level of hormones exceeded a certain threshold, it is still influenced by these factors in the same way as patients with suboptimal responses. BMI is another recognized factor that affects trigger response. Although it was controlled during subject enrollment in our study, significant differences in BMI were still observed between groups with varying hormone concentrations. Volume dilution of plasma concentration from body weight was considered as a possible mechanism for this association [29]. Compared to individuals with normal weight, overweight and obese people are more likely to have decreased post-trigger concentrations, which make them more prone to having low oocyte yields.

To explore the possible genetic factors for the patterns of post-trigger hormone levels, we detected polymorphisms of LHCGR N312S (rs2293275), which is located near a glycosylation site and affects ovarian sensitivity to gonadotropins with variations in the sequence. Heterozygous and homozygous N of these loci were previously found to be linked to a 2- to threefold increased risk of PCOS [31, 32], whereas in another population, a significant association of homozygous S and PCOS was demonstrated [33]. According to our results, the proportion of patients with the AG genotype was higher in the low LH group, and the post-trigger concentrations of LH and the ORR and MORR of these patients were lower than those of patients with the GG genotype after the GnRH-a trigger. These findings may provide a genetic explanation of the association between post-trigger LH (hCG) levels and oocyte yield, since both hCG and GnRH-a act on ovaries through the same receptor, LHCGR. In our study, no difference in the proportion of patients with PCOS among various hormone-level groups could be discerned probably due to the ethnic origin and the limited number of patients with PCOS. For instance, only one patient with the AA genotype made it difficult to analyse the impact of homozygous N. It should be noted that, as shown in Table 3, patients with AG and GG genotypes had comparable BMI, ovarian stimulation days and gonadotropin doses during the cycles with the same triggering, indicating that the post-trigger concentration of LH was affected by the genotype of LHCGRN 312S despite differences in BMI and gonadotropin use; that is, patients with the AG genotype are more likely to have low LH concentrations after the GnRH-a trigger. The usage of dual trigger with a GnRH agonist and a proper dosage of hCG could be a superior option for these patients because of the higher affinity and higher subsequent biological activity of hCG with the receptor. Based on our results, the oocyte yield and hormone concentrations after hCG trigger were not affected by the patient's genotype.

Although scholars have reported lower steroid levels in cases of GnRH-a triggers than in hCG trigger cycles [34], an equal number of oocytes between cycles with two triggers was claimed by most studies, in which hCG and GnRH-a were thought to have equivalent effects on follicular maturation [34–36]. In the present study, we found that the oocyte yield in patients with the AG genotype was significantly lower than that in patients with the GG genotype after the GnRH-a trigger, while the difference did not reach statistical significance in hCG trigger cycles, suggesting that oocyte recovery was affected by the genotypes of LHCGR beyond the triggers. Note that the GnRH-a trigger is normally used to prevent OHSS in cycles with excessive preovulation follicles [37], which probably counteracts the suboptimal ovarian response to the trigger. Consequently, the use of the “oocyte retrieval rate” rather than the number of oocytes would be advocated as a proper indicator to evaluate ovarian response to triggers and to predict oocyte recovery outcome. Additionally, the expected number of embryos in the GnRH-a trigger cycle is known to be more than that in the hCG trigger cycle. However, as shown in our study, the expected number of embryos was obtained from patients with the GG genotype between cycles of two trigger strategies (6.89 vs. 3.55), whereas the numerical advantage was lost in patients with the AG genotype (5.26 vs. 4.21). We speculated that the intensity of LH-like activity after triggering varied in patients with receptor polymorphisms; thus, limiting the use of the GnRH-a trigger to patients with the AG genotype would prevent the occurrence of low ORR and an unexpectedly low number of embryos.

## Conclusions

The present study demonstrates that serum LH concentration after the GnRH-a trigger is positively correlated with oocyte yield in antagonist cycles and that patients with the AG genotype of LHCGR rs2293275 in the Chinese Han population can have a lower oocyte yield than patients with the GG genotype using the GnRH-a trigger. The post-trigger hCG concentration was also significantly associated with oocyte yield, although the correlation was weak. This evidence highlights the role of LH-like activity in oocyte maturation, suggesting that oocyte yield increases with LH-like activity and can be regulated by receptor polymorphisms. These conclusions expand our understanding of endocrine changes during oocyte maturation and support the future design of individualized precise trigger protocols. Due to the limitation of sample size and the low number of patients with the AA genotype, further studies are needed to confirm these findings.

### Supplementary Information

**Additional file 1: Supplemental Table 1.** Descriptive date and genotype frequencies of 372 patients. **Supplemental Figure 1.** The body mass index (BMI), LH concentrations and number of high-quality embryos of patients grouped by oocyte retrieval rate (ORR) quartile in cycles with GnRH-a triggering.

## Data Availability

The datasets analysed during the current study are available from the corresponding author on reasonable request.
