# Supplementary material for: Post-trigger luteinizing hormone concentration to positively predict oocyte yield in the antagonist protocol and its association with genetic variants of LHCGR
Source: J Ovarian Res. 2023 Sep 11;16:189. doi: 10.1186/s13048-023-01271-6 (PMC10494325; doi:10.1186/s13048-023-01271-6)
Supplement: Supplementary file 1 — Additional file 1: Supplemental Table 1. Descriptive date and genotype frequencies of 372 patients. Supplemental Figure 1. The body mass index (BMI), LH concentrations and number of high-quality embryos of patients grouped by oocyte retrieval rate (ORR) quartile in cycles with GnRH-a triggering. [file 13048_2023_1271_MOESM1_ESM.docx]

Supplemental Table 1 Descriptive date and genotype frequencies of 372 patients.

| Characteristics | Post-trigger hormone concentration | | | P value |
| --- | --- | --- | --- | --- |
|  | Low group  (n=126) | Medium group  (n=123) | High group  (n=123) |  |
| Infertility causes |  | | | |
| Tubal | 71 (56.3) | 78 (63.4) | 82 (66.7) | 0.229 |
| PCOS | 37 (29.4) | 30 (24.4) | 30 (24.4) | 0.586 |
| Endometriosis | 8 (6.3) | 9 (7.3) | 8 (6.5) | 0.948 |
| Male | 29 (23.0) | 17 (13.8) | 21 (17.1) | 0.159 |
| Unexplained | 10 (7.9) | 11 (8.9) | 7 (5.7) | 0.613 |
| Trigger mode |  | | | |
| hCG | 70 (55.6) | 67 (54.5) | 68 (55.3) | 0.984 |
| GnRH-a | 56 (44.4)) | 56 (45.5) | 55 (44.7) | 0.984 |
| LHCGR rs2293275 genotype |  | | | |
| GG | 99 (78.6) | 107 (87.0) | 114 (92.7) | 0.005 |
| AG | 26 (20.6) | 16 (13.0) | 9 (7.3) | 0.009 |
| AA | 1 (0.8) | 0 (0.0) | 0 (0.0) | 0.376 |

Note: Data are presented as the numbers (percentage). P values are from the χ2 test or Fisher’s exact test. hCG, human chorionic gonadotropin; GnRH-a, gonadotropin-releasing hormone agonists; PCOS, polycystic ovary syndrome.

Supplemental Figure 1 The body mass index (BMI), LH concentrations and number of high-quality embryos of patients grouped by oocyte retrieval rate (ORR) quartile in cycles with GnRH-a triggering.


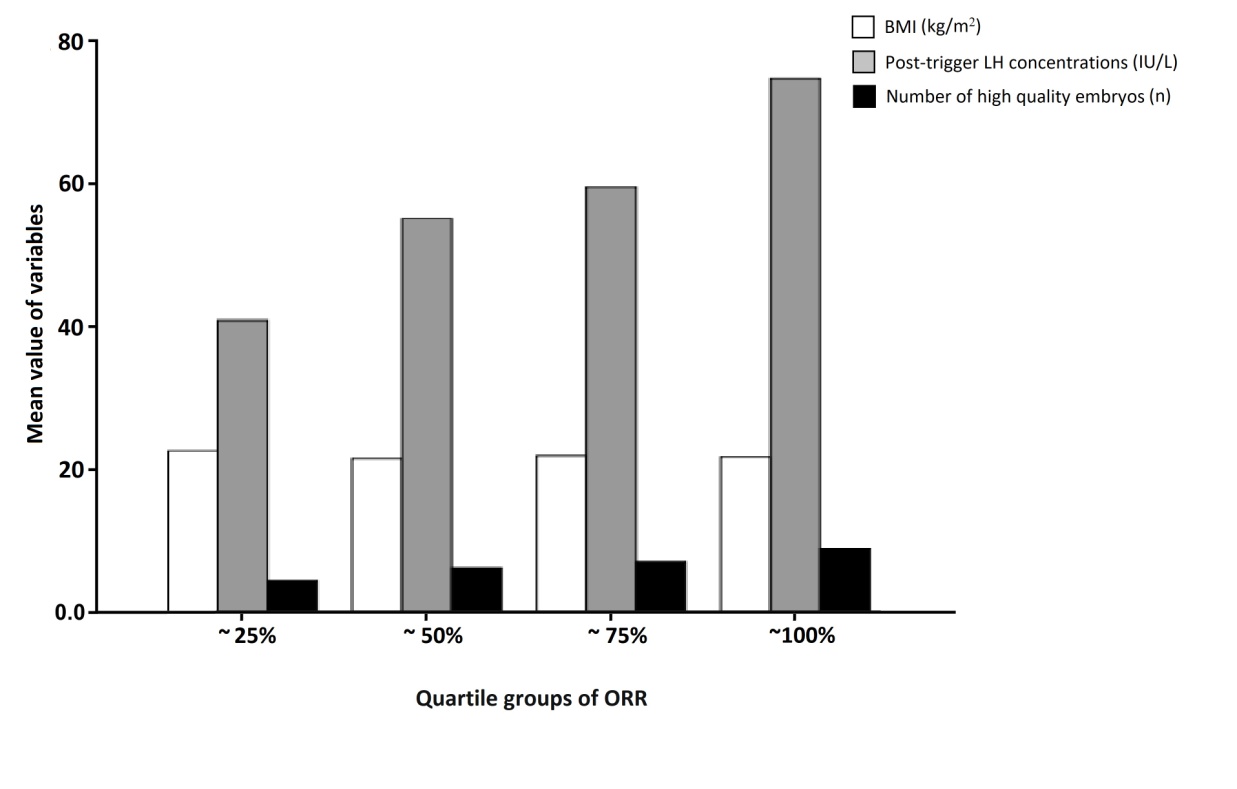


The post-trigger LH concentrations of each group were 41.30±16.35 IU/L, 55.74±23.76 IU/L, 60.22±20.45 IU/L and 75.66±33.26 IU/L, respectively. The number of high-quality embryos was 4.41±2.86, 6.14±3.72, 6.98±3.97 and 8.82±6.29, respectively. Analysis of variance showed significant differences in LH concentration (P<0.001) and number of high-quality embryos (P<0.001) among groups, while BMI was comparable (P=0.417).
